# Is There Pathological Uniformity between the Periphery and Center of a Gastrointestinal Stromal Tumor?

**DOI:** 10.3390/jcm10040687

**Published:** 2021-02-10

**Authors:** Seong Ji Choi, Kwan Hong Lee, Chan Kyoo Yoo, Jai Hoon Yoon, Ki Seok Jang, Youn Jeong Kim, Hang Lak Lee

**Affiliations:** 1 Department of Gastroenterology, Hanyang University Hospital, Seoul 04763, Korea; drcoolandy@gmail.com (S.J.C.); kwansover@naver.com (K.H.L.); camebulmyul@gmail.com (C.K.Y.); jaihoonyoon@hanyang.ac.kr (J.H.Y.); 2 Department of Pathology, College of Medicine, Hanyang University, Seoul 04763, Korea; medartisan@hanyang.ac.kr; 3 Department of Internal Medicine, College of Medicine, The Catholic University of Korea, Incheon ST. Mary’s Hospital, Incheon 21431, Korea

**Keywords:** gastric gastrointestinal stromal tumors, mitotic count, pathological uniformity

## Abstract

Background: Gastrointestinal stromal tumors (GISTs) are mesenchymal tumors and have some malignant potential. Mitotic count is important for predicting the malignant potential of GISTs. Proper treatment of GISTs requires accurate pathological diagnosis. In general, endoscopic ultrasound-guided fine-needle aspiration and deep biopsy are used for pathological diagnosis of GIST before making decisions about surgery. This study sought to evaluate the pathological uniformity of gastric GISTs for mitotic index of the center and periphery of the GIST. Methods: We retrospectively reviewed the data of 37 gastric GIST patients who underwent wedge resection at Hanyang University Hospital. We used Armed Forces Institute of Pathology criteria to classify gastric GISTs. To determine the pathological uniformity of gastric GISTs, we compared GIST risk stratification between the center and periphery of GISTs. Results: The mean size of GISTs was 3.56 ± 2.10 cm. Three lesions were located in the antrum, 11 in the fundus, 9 in the cardia, and 14 in the body. The mean age of patients was 58.65 ± 9.44 years; 18 patients were male and 19 were female. Thirty-five patients (94.6%) showed the same level of risk stratification between the center and periphery of gastric GISTs, while two patients (5.4%) presented different levels of risk between the two sites. No significant difference in mitotic count was observed between the two sites (kappa value = 0.863; *p* = 0.001). Conclusions: Mitotic index category (either more than five mitoses per high-power field or five or fewer mitoses per high-power field) of GISTs showed good concurrence between the center and periphery.

## 1. Introduction

Gastrointestinal stromal tumors (GISTs) are well-understood mesenchymal tumors that originate from the interstitial cells of Cajal known as intestinal pacemakers [1]. Because of improvements in diagnostic technique and increase in routine health care examinations, the incidence of GISTs is increasing in several countries [2,3,4]. GISTs may be asymptomatic and found incidentally; however, they are currently regarded as potentially malignant tumors, with about 10% to 30% progressing definitively to malignancy [5]. Risk stratification in patients with GISTs is important to determine treatment, follow-up strategies, and prognosis. There have been some prognostication systems introduced to predict malignant potential such as metastasis or recurrence, with several studies reporting that tumor size, mitotic index, primary tumor site, and tumor rupture are associated with prognosis in patients with GISTs [6,7,8,9]. According to the National Institutes of Health consensus criteria published in 2002, the most important prognostic factors of GISTs are mitotic index and tumor size [9]. Elsewhere, Armed Forces Institute of Pathology (AFIP) criteria considered tumor location to be a prognostic factor and, in cases of gastric GISTs, a tumor size of 10 cm or less and ≤5 mitoses per 50 high-power fields (HPFs) impart a low risk for metastasis, whereas those with >5 per 50 HPFs and tumors measuring greater than 5 cm in diameter have a high risk for metastasis [10]. Mitotic rate represents tumor cell proliferation; therefore, mitotic index has been suggested to be one of the most important factors to predict the behavior of cells in patients with GISTs [11]. However, mitosis identification must be conducted carefully because it can be subjective according to pathologist or measurement site, and it remains unknown where is the best place to measure mitotic index. In addition, endoscopic approaches such as endoscopic ultrasound (EUS)-guided fine-needle aspiration (FNA) or deep biopsy, which are traditionally considered the most established tissue sampling methods for gastric GISTs, would be confined to assessing peripheral lesions with small tissue volume because of their difficulty in approaching the tumor. It is not clear whether or not the preoperative pathologic diagnosis of gastric GISTs using endoscopy would accurately represent the whole mass. To our knowledge, no recent studies have commented on differences in mitotic index between the periphery and center of the tumor in patients with GISTs. When diagnosing GISTs of the stomach, it is important to know whether the biopsy findings can accurately diagnose the pathological findings of the entire GIST when performing histological confirmation through ultrasound or deep biopsy. Therefore, this study sought to confirm the pathological uniformity of the number of mitoses between the periphery and the center of the lesion in gastric GISTs.

## 2. Methods

### 2.1. Patients

A total of 37 patients who underwent curative surgical resection for gastric GISTs at Hanyang University Seoul Hospital between January 2008 and December 2014 was included. We reviewed the pathological and clinical data retrospectively. This study was approved by the Institutional Review Board of Hanyang University Seoul Hospital (no. 2015-02-004-001).

### 2.2. Surgical Resection

Surgical resection was performed in patients with gastric GISTs measuring larger than 2 cm with consideration of increasing tumor size; symptoms; physician opinion; patient preference; or EUS features indicating high risk of malignancy such as large size, irregular border, heterogeneous echogenicity, cystic spaces, or hyperechoic foci. Of 37 patients with gastric GISTs, 35 (94.6%) were treated by wedge resection, one underwent antrectomy with Billroth anastomosis, and one underwent total gastrectomy. A single experienced surgeon who worked at Hanyang University Hospital, Hanyang University College of Medicine performed all surgeries.

### 2.3. Pathological Diagnosis of Gastric GISTs

All slides were stained by hematoxylin and eosin to identify mitotic index. We counted mitotic figures by visual inspection with a 400× magnification. Pathological diagnosis of gastric GISTs was based on histopathology (i.e., epithelioid, spindle cell, mixed) and immunohistochemical analysis using CD117 (c-kit), CD34, S100, desmin, and smooth muscle actin antibodies.

### 2.4. Risk Stratification

**Figure 1:**
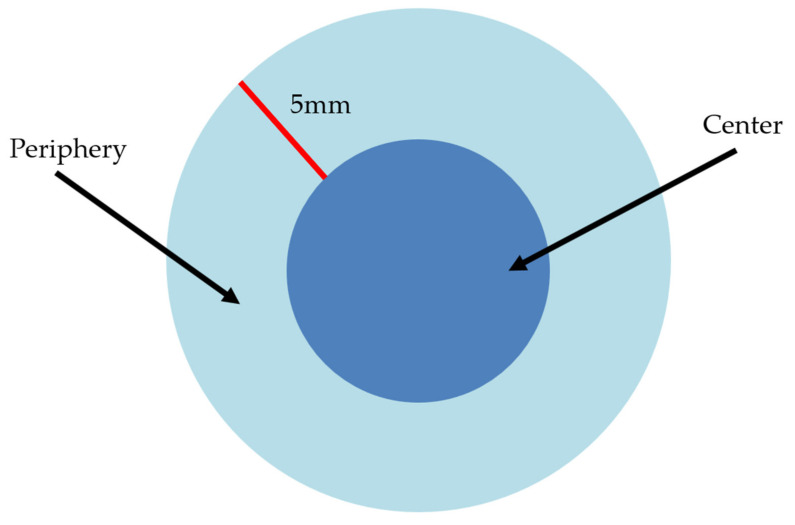
Schematic definitions of the center and periphery of a gastric gastrointestinal stromal tumors (GISTs) during pathological review.

**Figure 2:**
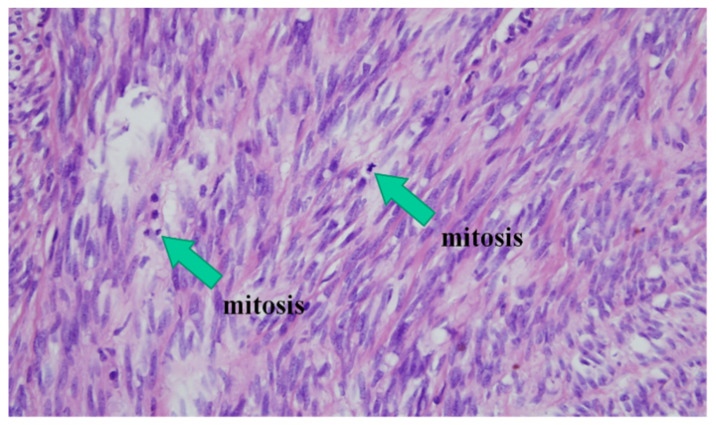
Mitosis in pathological findings. hematoxylin and eosin, original magnification, 400×.

The risk assessment of the tumors was conducted by applying the criteria of the AFIP. Patients were divided into four prognostic groups (very low, low, intermediate, and high risk) by tumor size, location, and mitotic rate to evaluate the likelihood of gastric GIST malignant behavior (Table 1) [8,10]. All slides were re-reviewed by a single pathologist specializing in gastrointestinal pathology. In clinically reported general pathology reports (primary reports), mitosis was counted in the most active cellular area of the pathologic specimen. GIST centers were defined as a part of the tumor starting at 5 mm from the surface of the tumor (Figure 1). We compared the level of risk stratification between the center and periphery of GIST masses. Mitotic index was counted under 50 HPFs and at three different sites within the tumor (Figure 1 and Figure 2).

### 2.5. Statistical Analysis

Continuous variables were expressed as mean ± standard deviation (SD), and categorical variables were expressed as frequency (%). Categorical data were analyzed using the Chi-square test with kappa values to compare tumor size and mitotic index between the periphery and center. *P*-values <0.05 were considered statistically significant. All analyses used the Statistical Package for the Social Sciences version 18.0 (IBM Corp., Armonk, NY, USA).

## 3. Results

### 3.1. Clinical Features of Gastric GISTs

Table 2 presents the baseline characteristics and endoscopic findings of 37 gastric GIST patients with a 1:1.06 male-to-female ratio and a mean age of 58.65 ± 9.44 years (range: 40–76 years). The mean tumor size was 3.56 ± 2.10 (range: 2.0–11.5) cm, and 32 (86.5%) measured between 2 and 5 cm. Of these, 27 cases (72.9%) were found incidentally on endoscopy during a health care examination and, among 10 symptomatic cases, nausea and anorexia (*n* = 5) were the most common symptoms, followed by abdominal discomfort (*n* = 4) and gastrointestinal bleeding (*n* = 1). Gastric body was the most common involved site (*n* = 14; 37.8%), followed by fundus (*n* = 11; 29.7%), cardia (*n* = 9; 24.3%), and antrum (*n* = 3; 8.1%). Endoscopic ultrasonography revealed findings of 100% hypoechoic lesions and 64.9% (*n* = 24) with a heterogeneous echo pattern. Positive CD34, desmin, SMA, and S-100 expression was detected in 94.6%, 2.7%, 10.8%, and 5.4% of the GISTs, respectively. One patient experienced recurrence.

### 3.2. Primary Pathologic Reports

The most prominent histologic type was spindle-shaped (*n* = 33; 89.2%), followed by mixed epithelioid and spindle type (*n* = 4; 10.8%). The mitotic count was ≤5/50 HPFs in 29 cases (78.4%), 6–10/50 HPF in 3 cases (8.1%), and >10/HPF in 5 cases (13.5%). In the primary report, the original risk stratification by AFIP criteria suggested that 3 cases were at no risk, 22 were at very low risk, 1 was at low risk, 8 were at moderate risk, and 3 were at high risk (Table 2).

### 3.3. Comparison of Risk Stratification between the Periphery and Center of Gastric GISTs

**Figure 3:**
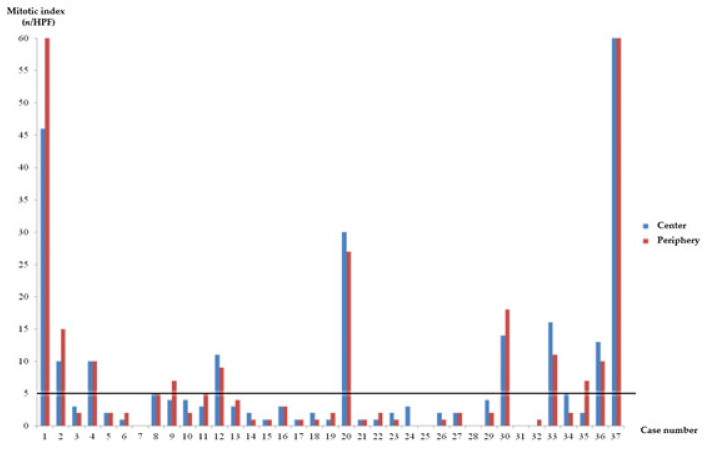
Mitotic index agreement between the center and the periphery. HPF, High-Power Field.

Table 3 summarizes the EUS findings and risk stratification according to site of mitotic index analysis of the 37 cases included in this study. Among 37 gastric GISTs, 94.6% (*n* = 35) showed the same level of risk stratification for the center and the periphery. Only two (5.4%) cases had different risk stratification outcomes between the two sites. One case (case 9 in Table 3) was categorized as moderate risk in the original pathologic report but as very low risk and moderate risk according to assessment of the center and periphery of the gastric GIST, respectively. The other case (case 35) was categorized as moderate risk in the primary pathologic report but very low risk and moderate risk according to assessment of the center and periphery regions of the gastric GIST, respectively. Figure 3 showed the comparison of mitotic index between center and periphery site. Overall, mitotic index category (>5/HPF, ≤5/HPF) of GISTs showed good agreement between the center and the periphery (*K* = 0.74; *p* < 0.001).

## 4. Discussion

In our study, mitotic indices of the periphery and center of gastric GISTs showed good concurrence. To the best of our knowledge, this study is the first to compare mitotic count between the center and periphery of gastric GISTs. Yasui et al. reported the heterogeneity of gastric GIST lesions using the MIB-1 index, and the discrepancy in the result might be due to the difference of the patient characteristics included: our study included only resectable gastric GISTs, but patients included in the study by Yasui et al. showed much larger mean diameter of GIST (6.9 ± 2.73 vs. 3.56 ± 2.10) [12]. This difference in result might suggests that the GIST could become more heterogenous as the size of the lesion increases or during the process of metastasis, but further research is needed. Usually gastric GISTs sized more than 2 cm are preferred to be removed [1]. However, if the patient had comorbidity or high risk of operation, accurate evaluation of risk classification is helpful to decide treatment plan. According to our results, since the heterogenicity of the central and peripheral is not significant, we think the biopsy at peripheral could assess risk classification.

GISTs are the most common mesenchymal tumors of the gastrointestinal tract and high incidence rates of 19 to 22 cases per one million inhabitants have been reported in Korea, China, and Norway [13]. While most GISTs are found in the stomach, 20% to 30% are present in the small intestine, 5% to 15% present in the colon, and less than 5% present in the esophagus and other regions [8,14,15,16]. Although the only principal treatment of localized GISTs is surgical resection with negative margins, there is a risk for recurrence or metastasis; one cohort study reported that 29.4% of cases showed recurrence or metastasis following curative resection of gastric GISTs during a median 31.95 months of follow-up [17]. Our study revealed a lower recurrence rate of 2.7%, which may be the result of bias due to our small sample size and inclusion of only patients with gastric GISTs who underwent curative surgical resection.

Contour maps based on a pooled population-based study of GISTs who received no adjuvant therapy revealed that tumor size, mitosis count, primary tumor site, and rupture can predict recurrence at 10 years after surgery [18]. Among these factors, high mitotic index is one important risk factor that has been included among various prognostic criteria for GISTs including the National Institutes of Health (NIH) classification, the modified NIH classification, and the AFIP classification [7,9,10]. In order to raise the survival rate in the resectable GIST tumor, for large tumor size and large mitotic count, tyrosine kinase inhibitors can be used as adjuvant therapy. Therefore, it is important to evaluate the mitotic count before treatment [19]. Usually, the number of mitoses is counted among 50 consecutive HPFs from the most cellularly dense areas (area of an individual field: 0.2 mm2); however, it is sometimes difficult to complete this because of subjectivity in relation to the pathologist or resection site involved. While preoperative histological diagnosis using EUS-guided FNA or deep biopsy is necessary to guide adjuvant therapy or patient counseling, EUS-guided biopsy presents the limitation of small tissue volume. In our study, only 5.4% of cases showed different risk categories for the center and peripheral sites, and there was good concurrence of mitotic count on the basis of 5/HPF between the center and periphery. Surprisingly, the original data largely matched in terms of risk category with the periphery but the not center results, suggesting that peripheral small-tissue sampling through EUS-guided FNA or deep biopsy may be enough to assess mitosis among patients with gastric GISTs. Our study had several limitations. First, this study had a retrospective design and a small number of patients. Second, we did not analyze the pathologic results using EUS-guided FNA or deep biopsy because preoperative biopsy tends not to be performed when GISTs are strongly suspected and surgery is under consideration. Third, our study only included gastric GIST that is usually rather low risk of malignancy. Therefore, our finding may be difficult to apply to other tumors such as high risk tumors, or other sites of GIST lesions.

## 5. Conclusions

Our data indicated that mitotic indices of the periphery and center of gastric GISTs showed good concurrence, and the periphery was the same risk category as the original data. Biopsy using endoscopic approaches is likely to represent whole gastric GIST mass, although there is some degree of limitation because of the limited tissue volume and difficulty with approaching the tumor.

## Figures and Tables

**Table 1:** Risk stratification of gastric gastrointestinal stromal tumors (GISTs) (risk for progressive disease) ^†^.

Risk of Progression (%)	Mitotic Index (High-Power Field)	Tumor Size (cm)
None (0%)	≤5/50	≤2
None (0%, small cases) **	>5/50	≤2
Very low (1.9%)	≤5/50	>2, ≤5
Low (3.6%)	≤5/50	>5, ≤10
Moderate (12%)	≤5/50	>10
(16%)	>5/50	>2, ≤5
High (55%)	>5/50	>5, ≤10
(86%)	>5/50	>10

^†^ modified from Miettinen et al [1]; ** Small number of cases.

**Table 2:** Baseline characteristics of enrolled patients.

	Gastric GISTs (*n* = 37)
Age, years	58.65 ± 9.44 (40–76)
Sex (M/F)	18(48.6%)/19 (51.4%)
Weight (kg)	66.62 ± 10.08 (46–86)
Height (meters)	1.62 ± 0.92 (1.44–1.88)
BMI (kg/m^2^)	25.30 ± 2.92 (19.40–30.86)
Symptomatic GISTs, *n* (%)	10 (27%)
Abdominal pain	4 (10.8%)
Hematemesis	1 (2.7%)
Nausea, anorexia	5 (13.5%)
Incidental GISTs, *n* (%)	27 (73%)
DM, *n* (%)	10 (27%)
Hypertension, *n* (%)	19 (51.4%)
Hepatitis, *n* (%)	1 (2.7%)
Malignancy, *n* (%)	2 (5.4%)
Size (cm)	3.56 ± 2.10 (2.0–11.5)
Location, *n* (%)	
Antrum	3 (8.1%)
Body	14 (37.8%)
Fundus	11 (29.7%)
Cardia	9 (24.3%)
EUS findings	
Echogenicity, *n* (%)	
Hypoechoic/hyperechoic	37 (100%)/0 (0%)
Homogeneity, *n* (%)	
Homogenous	13 (35.1%)
Heterogenous	24 (64.9%)
Cystic change	
Negative/positive	20 (54.1%)/17 (45.9%)
Ulcer of gastric mucosa	6 (16.2%)
Immunohistochemistry, *n* (%)	
c-Kit	37 (100%)
CD34	35 (94.6%)
Desmin	1 (2.7%)
SMA	4 (10.8%)
S-100	2 (5.4%)
Primary risk stratification	
No risk	3 (8.1%)
Very Low	22 (59.65%)
Low	1 (2.7%)
Moderate	8 (21.6%)
High	3 (8.1%)
Postoperative chemotherapy, *n* (%)	4 (10.8%)
Recurrence, *n* (%)	1 (2.7%)

Values are presented as mean ± standard deviation (range) or number (%).

**Table 3:** Endoscopic ultrasound and pathological findings for total 37 cases.

					EUS Findings							Pathologic Findings			
Case	Gender	Age (range)	Location	Size (cm)	Layer	Echogenecity	Heterogeneity	Cystic change	Size (cm)	Original (*n*/HPF)	Center (*n*/HPF)	Periphery (*n*/HPF)	Original (risk)	Center (risk)	Periphery (risk)
1	1	60–70	Fundus	4	4th	Hypo	Homo	Present	3.5	104	46	120	Moderate	Moderate	Moderate
2	2	50–60	Cardia	3.6	4th	Hypo	Hetero	Present	5.3	8	10	15	High	High	High
3	1	50–60	Antrum	2.6	4th	Hypo	Hetero	Present	2.7	3	3	2	Very low	Very low	Very low
4	2	50–60	Fundus	3.1	4th	Hypo	Hetero	Present	3.1	8	10	10	Moderate	Moderate	Moderate
5	2	60–708	Fundus	4.2	4th	Hypo	Hetero	Absent	5.5	1	2	2	Low	Low	Low
6	1	50–60	Body	3.4	4th	Hypo	Hetero	Absent	2.7	1	1	2	Very low	Very low	Very low
7	1	60–70	Antrum	2.4	4th	Hypo	Homo	Absent	2.2	2	0	0	Very low	Very low	Very low
8	2	60–70	Body	2.2	4th	Hypo	Homo	Absent	3	2	5	5	Very low	Very low	Very low
9	1	50–60	Body	3.2	4th	Hypo	Hetero	Absent	3	7	4	7	Moderate	Very low	Moderate
10	1	70–80	Fundus	4.5	4th	Hypo	Homo	Present	4.5	2	4	2	Very low	Very low	Very low
11	1	70–80	Body	2.3	4th	Hypo	Homo	Absent	3	1	3	5	Very low	Very low	Very low
12	2	60–70	Cardia	2.4	4th	Hypo	Homo	Present	2.5	7	11	9	Moderate	Moderate	Moderate
13	1	60–70	Antrum	2.1	4th	Hypo	Hetero	Present	2	3	3	4	No risk	No risk	No risk
14	2	60–70	Fundus	2.5	3rd	Hypo	Hetero	Absent	2	0	2	1	No risk	No risk	No risk
15	1	40–50	Cardia	2.5	4th	Hypo	Homo	Absent	3	0	1	1	Very low	Very low	Very low
16	2	60–70	Body	2.7	4th	Hypo	Hetero	Absent	3	5	3	3	Very low	Very low	Very low
17	2	50–60	Body	3.1	4th	Hypo	Homo	Absent	2.5	1	1	1	Very low	Very low	Very low
18	2	40–50	Body	2.8	4th	Hypo	Hetero	Absent	3	3	2	1	Very low	Very low	Very low
19	1	40–50	Body	4.2	4th	Hypo	Hetero	Absent	4	2	1	2	Very low	Very low	Very low
20	1	60–70	Fundus	4.1	4th	Hypo	Hetero	Absent	5	50	30	27	Moderate	Moderate	Moderate
21	1	50–60	Cardia	4	4th	Hypo	Homo	Absent	4	3	1	1	Very low	Very low	Very low
22	2	60–70	Cardia	3	4th	Hypo	Hetero	Present	3.3	2	1	2	Very low	Very low	Very low
23	2	50–60	Fundus	2.5	3rd	Hypo	Hetero	Absent	2.7	3	2	1	Very low	Very low	Very low
24	1	50–60	Body	4	4th	Hypo	Homo	Absent	3.5	1	3	0	Very low	Very low	Very low
25	1	40–50	Cardia	2.3	4th	Hypo	Hetero	Present	2.2	2	0	0	Very low	Very low	Very low
26	2	40–50	Cardia	4.7	4th	Hypo	Hetero	Present	4.5	4	2	1	Very low	Very low	Very low
27	2	70–80	Body	2.2	4th	Hypo	Homo	Absent	2	1	2	2	No risk	No risk	No risk
28	2	60–70	Body	11	4th	Hypo	Hetero	Present	10.5	1	0	0	Moderate	Moderate	Moderate
29	2	40–50	Cardia	4.5	4th	Hypo	Hetero	Present	3.8	2	4	2	Very low	Very low	Very low
30	2	50–60	Fundus	11	4th	Hypo	Hetero	Present	11.5	16	14	18	High	High	High
31	2	50–60	Fundus	3.5	4th	Hypo	Hetero	Absent	2.3	0	0	0	Very low	Very low	Very low
32	1	60–70	Fundus	3	4th	Hypo	Hetero	Absent	2.2	0	0	1	Very low	Very low	Very low
33	1	70–80	Cardia	2	4th	Hypo	Hetero	Present	2.1	7	16	11	Moderate	Moderate	Moderate
34	2	60–70	Body	2	4th	Hypo	Hetero	Present	2.2	3	5	2	Very low	Very low	Very low
35	2	60–70	Body	4.2	4th	Hypo	Hetero	Present	3.7	7	2	7	Moderate	Very low	Moderate
36	1	50–60	Body	2.2	4th	Hypo	Homo	Present	2.3	11	13	10	Moderate	Moderate	Moderate
37	1	40–50	Fundus	4.5	4th	Hypo	Hetero	Absent	5.3	118	160	125	High	High	High

## Data Availability

The datasets used and/or analyzed are available from the corresponding author on reasonable request. The data are not publicly available due to ethical and privacy restrictions.
